# Influence of photoperiods on the growth rate and biomass productivity of green microalgae

**DOI:** 10.1007/s00449-013-1044-x

**Published:** 2013-09-14

**Authors:** Izabela Krzemińska, Barbara Pawlik-Skowrońska, Magdalena Trzcińska, Jerzy Tys

**Affiliations:** 1 Institute of Agrophysics, Polish Academy of Sciences, Doświadczalna 4, 20-290 Lublin, Poland; 2 Department of Hydrobiology, University of Life Sciences in Lublin, Dobrzańskiego 37, 20-262 Lublin, Poland; 3 Polish Academy of Sciences Centre for Ecological Research, Experimental Station, Niecała 18, 20-080 Lublin, Poland

**Keywords:** Green microalgae, Light regime, Biomass productivity, Autotrophic cultivation

## Abstract

The effect of different photoperiods: 24 h illumination and a 12:12-h light/dark (12L:12D) cycle on the growth rate and biomass productivity was studied in five algal species: *Neochloris* *conjuncta*, *Neochloris* *terrestris*, *Neochloris* *texensis*, *Botryococcus* *braunii* and *Scenedesmus obliquus*. The green microalgae examined differ in the reproduction mode. Continuous illumination stimulated the growth of *B.* *braunii* and *S.* *obliquus* more effectively than the growth of the microalgal species from the genus *Neochloris*. However, under shorter duration of light of the same intensity (12L:12D cycle), the growth of all the three species of *Neochloris* was stimulated. Under continuous illumination, the specific growth rate in the first phase of *B.* *braunii* and *S.* *obliquus* cultures was higher than the growth rate of *Neochloris*, whereas under the 12L:12D cycle, the specific growth rate of all the three *Neochloris* species was generally higher than that in *B.* *braunii* and *S.* *obliquus*. As a result, the light regime influenced algal biomass productivity differently. The maximum biomass productivity was obtained in *B. braunii* and *S. obliquus* cultures carried out at continuous illumination. All the *Neochloris* species produced biomass more efficiently at the 12L:12D cycle, which was two–threefold higher than that of *B. braunii* and *S. obliquus*. The unicellular species of the green microalgae from the genus *Neochloris*, examined for the first time in this study, are promising prospective objects for algal biotechnology.

## Introduction

The increasing demand for alternative energy sources increases the interest in biofuel production. One of the research objectives undertaken is biofuel production from microalgal biomass [1, 2]. Microalgal biomass may be converted into a variety of biofuels. Biochemical conversion of biomass through the fermentation process yields biomethane and bioethanol, thermochemical conversion results in bio-oil production, and transesterification of lipids yields a biodiesel product. Some algal species have the ability to produce hydrogen through photobiological processes. The advantage of microalgal biomass over traditional energy-plant sources of biomass is the rapid growth rate of microalgae and accumulation of substantial amounts of carbohydrates and fats [3].

Microalgae are of interest for biotechnological purposes because of the ability to accumulate and store secondary metabolites and to efficiently produce functionally active proteins. The biotechnological potential of microalgae is related to the fact that their biomass contains valuable components, including lipids, starch, and alkanes [4]. Therefore, microalgal biomass is considered as one of the promising feedstock for biofuels and chemicals. Microalgae are used in the production of diverse components, e.g., dyes, antioxidants, gelling agents, emulsifiers, aminoacids, and fatty acids omega 3 and 6 [5]. Microalgal pigments and proteins have great potential for medical application [6]. Microalgal hydrocarbons and polysaccharides can be converted into ethylene, propylene, adipic acid, and furabics [4].

Environmental factors that exert an impact on microalgal growth include temperature, pH, salinity, inorganic carbon availability, and light. Light is one of the key factors that control the course of physiological processes in microalgae. The quantity and quality of light determines the amount of available energy that is indispensable for the photosynthetic process. Equally important is the dark/light regime, which influences algal growth and biomass production. In the natural environment, light intensity undergoes continuous changes, and the light regimen is not constant [7]. Changes in light quantity induce alterations in the biochemical composition of microalgae. Increased frequencies of the light/dark cycles may considerably enhance productivity and photosynthetic efficiency [8]. Recently, investigations concerning the influence of the photoperiod on the biomass yield in several freshwater and marine microalgae, e.g., *Chlamydomonas* *reinhardtii*, *Chlorella* *sorokiniana*, *Dunaliella* *tertiolecta* [9], *Chlorella* *vulgaris* [7], have been carried out.

Individual algal species differ in terms of nutritional and light requirements, life cycles, and modes of reproduction. Therefore, culture conditions have a substantial effect on the algal proliferation rate and biomass production.

The aim of the study was to compare the growth rate and biomass productivity of five chlorophyte species of different reproduction modes: *Scenedesmus* *obliquus*, *Botryococcus* *braunii*, *Neochloris* *conjuncta*, *Neochloris* *terrestris* and *Neochloris* *texensis*. The three latter species from the *Neochloris* genus have not been studied yet.

## Materials and methods

Strains of the green microalgae *B.* *braunii* SAG 30.81, *S.* *obliquus* SAG 276-3a, *N.* *conjuncta* SAG 78.80, *N.* *terrestris* UTEX B. 947, and *N.* *texensis* SAG 99.80 originating from the SAG Culture Collection of Algae and the UTEX Culture Collection of Algae were inoculated from solid into sterile liquid Kessler’s medium to obtain a sufficiently large quantity of algal biomass required for the experiments. Preliminary semi-continuous cultures were run under light (Osram L58W/765 cool daylight) and temperature 24 ± 1 °C for 60 days until biomass suitable for the experiments was obtained. The intensity of photosynthetically active light (PPFD) was 60 μmol m^−2^ s^−1^.

The biomass obtained was used (1) for determination of the relationship curves between the optical density of algal culture measured with the spectrophotometric method (Unicam Helios, UK) at the 650-nm wavelength and the dry weight (determined with the weighing method) of algae growing under the conditions specified above, and (2) as an inoculum for the growth experiments.

## Phototrophic cultivation

The growth of the stationary cultures of the individual algal species on the sterile liquid Kessler’s medium under the aforementioned conditions was monitored for 10 days at constant 24-h illumination (experimental variant I) or at a 12:12-h light:dark cycle (variant II). The initial dry weight content for cultures of each alga was 20 mg dry weight/L and the initial optical densities (OD_650_) for the cultures of particular species were as follows: *B.* *braunii* 0.031, *S.* *obliquus* 0.049, *N.* *conjuncta* 0.025, *N.* *terrestris* 0.032, and *N.* *texensis* 0.037. The medium used in this study contained: KNO_3_ 0.81 g, NaCl 0.47 g, NaH_2_PO_4_·2H_2_O 0.47 g, Na_2_HPO_4_·12H_2_O 0.36 g, MgSO_4_·7H_2_O 0.25 g, CaCl_2_·2H_2_O 0.014 g, FeSO_4_·H_2_O 0.006 g, MnCl_2_·4H_2_O 0.0005 g, H_3_BO_3_ 0.0005 g, ZnSO_4_·7H_2_O 0.0002 g, ZnSO_4_·7H_2_O 0.0002 g, (NH_4_)_6_Mo_7_O_24_·4H_2_O 0.00002 g, EDTA (Titriplex III Merck) 0.008 g/L, pH 7.0. The cultures were mixed by means of sterile air.

The growth of each culture was monitored daily for 10 days by spectrophotometric measurements of the optical density OD_650_, typical for live cells. Dry weight (DW) of algal biomass was determined after overnight drying at 90 °C.

A good linear relationship was found between the algal dry weight and the optical density (OD_650_) of the cultures. The correlation coefficients *R* ^2^ were close to the value of 1.0 (0.9991–0.9999) in the case of the algal strains from the genus *Neochloris*; they were only slightly lower (0.9965–0.9982) for the other strains. This allowed monitoring the changes in the algal growth and biomass in the liquid cultures with the use of the spectrophotometric method. All the experiments and determination were performed in triplicate.

Based on the curves of the correlations between OD_650_ and dry weight, the algal growth curves, biomass doubling time, changes in the specific growth rate in different culture phases (0–3 days, 3–10 days), and biomass productivity (after 10 days of cultivation) were determined. The specific growth rate of the microalgae was calculated using the equation *μ* = ln(N_2_/N_1_)/(*t*_2_ − *t*_1_), where *μ* is the specific growth rate, and N_1_ and N_2_ are the biomass at time 1 (*t*_1_) and time 2 (*t*_2_), respectively.

## Results

**Fig. 1:**
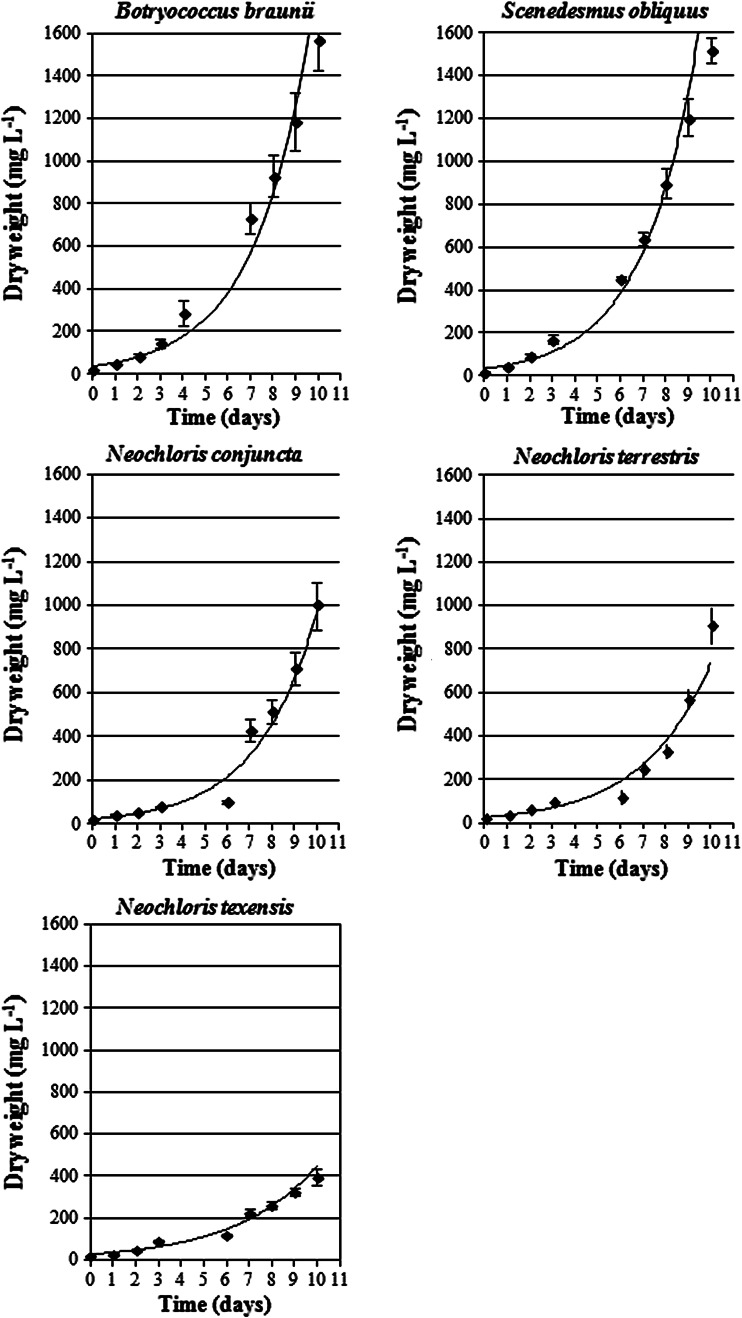
Comparison of growth of five green microalgae under continuous illumination. Data are expressed as mean ± SD, *n* = 3

**Fig. 2:**
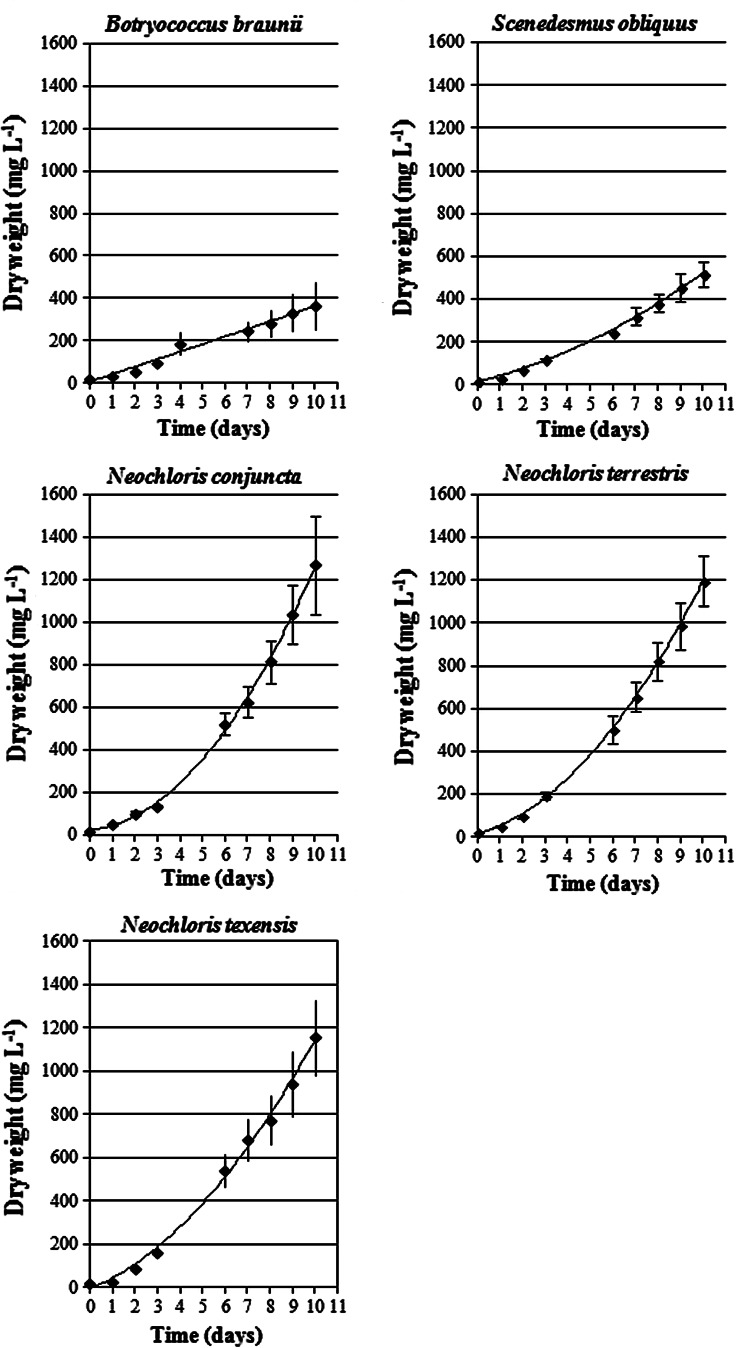
Growth curves of green microalgae under the 12L:12D cycle. Data are expressed as mean ± SD, *n* = 3

**Fig. 3:**
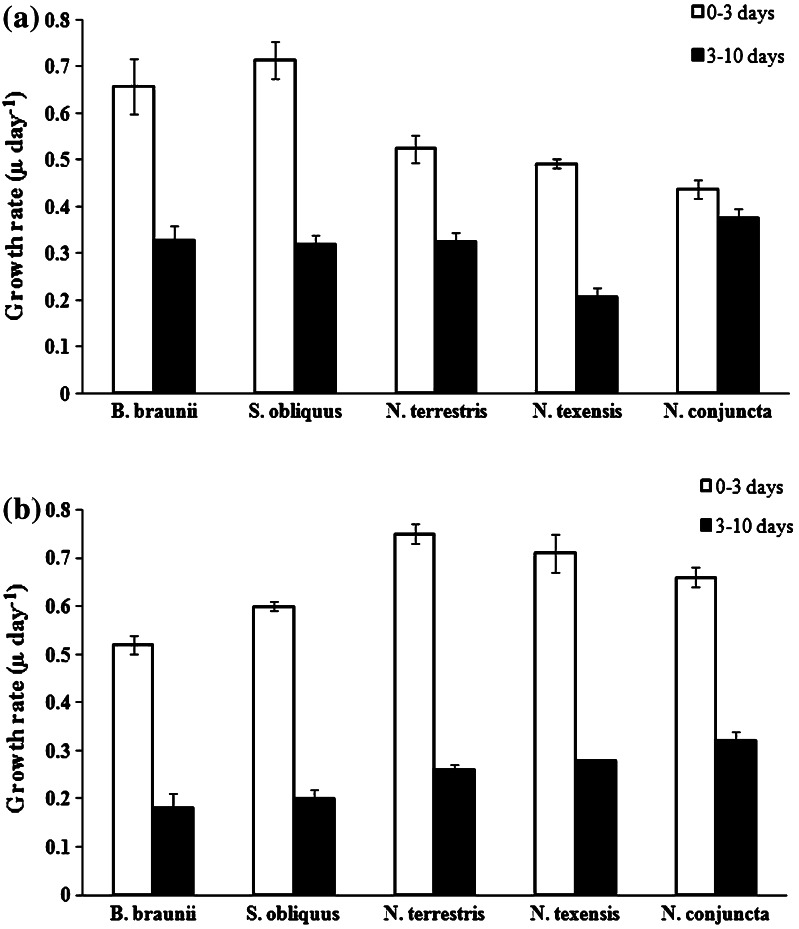
Specific growth rate in the green microalgae within 2 phases (0–3 days and 3–10 days) of culture under various photoperiods. **a** Continuous light; **b** 12L:12D cycle

The culture growth of the examined algal species differed and was dependent on the photoperiod applied (Figs. 1, 2). Continuous illumination stimulated the growth of *B.* *braunii* and *S.* *obliquus* more efficiently than the growth of the microalgal strains from the genus *Neochloris* (Fig. 1). In contrast, shorter duration of light (12L:12D photoperiod) led to increased growth of the three *Neochloris* species (Fig. 2). The effect of the light regime on the specific growth rate assessed in two phases: days 0–3 and days 3–10 of the culture is shown in Fig. 3. Under continuous illumination (Fig. 3a), the specific growth rate of *B.* *braunii* and *S.* *obliquus* in the first growth phase (up to 3 days) was generally higher (*μ* = 0.66 and 0.71 day^−1^, respectively) than that of the other strains (*N.* *terrestris*: *μ* = 0.52 day^−1^, *N. texensis*: *μ* = 0.49 day^−1^, *N.* *conjuncta*: *μ* = 0.44 day^−1^). The use of the more energy-efficient (in economic terms) 12L:12D cycle (Fig. 3 b) resulted in a decrease in the growth rate of *B.* *braunii* and *S.* *obliquus,* which was then lower than the growth rate of the three *Neochloris* species examined. At the 12L:12D photoperiod, the growth rate in the first phase of the culture (0–3 days) in all the *Neochloris* strains analyzed (*N.* *terrestris*: *μ* = 0.75 day^−1^, *N.* *texensis*: *μ* = 0.71 day^−1^, *N.* *conjuncta*: *μ* = 0.66 day^−1^) was generally higher than that in *B.* *braunii* and *S.* *obliquus*. In the second culture phase (3–10 days), the 12L:12D light regime also supported the higher growth rate of *Neochloris* than that of the other two algal species. The effect of the photoperiod on the biomass doubling time is summarized in Table 1. A comparison of all the species tested under continuous illumination revealed the shortest biomass doubling time for *B.* *braunii* (18.7 h, at continuous illumination) and for *N.* *conjuncta* (17.6 h) as well as *N.* *terrestris* (19.7 h) under the 12L:12D cycle. *B.* *braunii* and *S. obliquus* were characterized by a much shorter biomass doubling time in the constantly illuminated cultures than under the 12L:12D cycle. In turn, *N.* *conjuncta* and *N.* *terrestris* exhibited a considerably shorter biomass doubling time under the 12L:12D cycle than under continuous light. The different photoperiods did not influence essentially the doubling time of *N.* *texensis*.

**Table 1:** Biomass doubling time (h) in algal cultures growing at different photoperiods

Algal species	Photoperiod	
	24 h light	12 h light:12 h dark
*B. braunii*	18.7 (±0.55)	36.5 (±1.43)
*S. obliquus*	22.2 (±0.26)	28.0 (±0.57)
*N. conjuncta*	24.9 (±2.12)	17.6 (±1.14)
*N. terrestris*	27.8 (±2.17)	19.7 (±0.59)
*N. texensis*	30.9 (±1.97)	32.4 (±0.78)

Data are expressed as mean ± SD, *n*=3

The light regime had an essential effect on the microalgal biomass productivity. The productivity of the individual strains was correlated with the photoperiod applied (Table 2). The maximum biomass productivity was obtained in *B.* *braunii* and *S.* *obliquus* (0.155 and 0.150 g L^−1^ day^−1^, respectively) cultured under the continuous light conditions. All the *Neochloris* species grew more efficiently at the 12L:12D cycle than under continuous illumination and the biomass productivity of all the three *Neochloris* species was even two–threefold higher (0.114–0.125 g L^−1^ day^−1^) than that of *B.* *braunii* and *S.* *obliquus* (0.034 and 0.050 g L^−1^ day^−1^, respectively).

**Table 2:** Biomass productivity (g L^−1^ day^−1^)

Algal species	Photoperiod	
	24 h light	12 h light:12 h dark
*B. braunii*	0.155 (±0.014)	0.034 (±0.011)
*S. obliquus*	0.150 (±0.006)	0.050 (±0.006)
*N. conjuncta*	0.098 (±0.011)	0.125 (±0.023)
*N. terrestris*	0.089 (±0.008)	0.117 (±0.012)
*N. texensis*	0.037 (±0.004)	0.114 (±0.017)

Data are expressed as mean ± SD, *n*=3

## Discussion

Light intensity and photoperiod is one of the most important factors influencing the growth rate and biomass composition and, hence, production of high-value microalgal products in a wide range of algal species [7, 10–14]. Alterations in the photoperiod induce changes in the total protein, pigment and fatty acid content in *C.* *vulgaris* [13]; the growth and lipid production in *Porphyridium* *cruentum* [12] and *B.* *braunii* [15]; cell density, the cell growth rate and total lipid content in *Nannochloropsis* sp. [14]; biomass production and utilization of nutrients (nitrate and phosphate) by *Tetraselmis chui* [11] and biomass production in cyanobacterium *Aphanothece* [21].

The photoperiod is also important in terms of the economic aspect if algal biomass is produced with a supply of light from artificial sources. There are several reports on the influence of the photoperiod on the productivity and growth rate of *C.* *vulgaris* [7] and *Dunaliella* spp. [9], on the biomass concentration of *B.* *braunii* [15] and biomass growth of *S.* *obliquus* [16]. However, there are many other algal species that are potentially used for biomass production. No study on the unicellular green microalgae *Neochloris* spp. (except from *N. oleoabundans*) has been reported up to date. As shown by our results, the *Neochloris* coccoid microalgae display biomass productivity comparable to the widely described *B.* *braunii* and *S.* *obliquus* but at a lower “light energy input”, thus making them promising biotechnological objects. Recently, Khoeyi et al. [7] have reported that the light regime is an important factor controlling the biomass production of *C*. *vulgaris*. Longer duration of light resulted in increased biomass of *C*. *vulgaris* at different light intensities, and the increased specific growth rate was associated with an increase in light duration. These results are similar to these obtained in the present study for *B.* *braunii* and *S.* *obliquus*, for which the increased duration of light improved the specific growth rate. As shown in Fig. 3, the maximum specific growth rate was found for *B.* *braunii* and *S.* *obliquus* (0.64 and 0.71 μ day^−1^, respectively) under the 24-h light regime. The present study has shown a 4.5-fold higher (0.155 vs. 0.034 g L^−1^ day^−1^) biomass productivity of *B.* *braunii* under the continuous illumination in comparison with the 12L:12D cycle. This result is comparable to that obtained by Ruangsomboon [15], who reported that the total biomass concentration in *B.* *braunii* was 1.91 ± 0.24 g L^−1^ under a 24:0-light cycle, which was four times higher than the biomass obtained under the 12L:12D cycle. The biomass productivity of *B.* *braunii* and *S.* *obliquus* obtained under continuous illumination (0.155 and 0.150 g L^−1^ day^−1^ respectively) was slightly lower than the productivity of some *Chlorella* spp. The biomass productivity of different *Chlorella* strains ranged 0.18–0.34 g L^−1^ day^−1^ [17, 18]. Kim et al. [19] reported that the specific growth rate and biomass productivity of *Chlorella* sp. under a CO_2_ concentration 0.04 % and 100 μmol m^−2^ s^−2^ were 0.50 μ day^−1^ and 0.24 g L^−1^ day^−1^, respectively. The productivity of *C.* *vulgaris* [7] under limited time of illumination (12L:12D) and very similar light intensity (62.5 μmol m^−2^ s^−1^) was as such in *B.* *braunii* and *S.* *obliquus* (at 24 h illumination) in our study. Higher light intensity decreased *Chlorella* productivity. Ho et al. [20] reported that the biomass productivity of six tested *S.* *obliquus* strains ranged from 0.217 ± 0.02 g L^−1^ day^−1^ to 0.441 ± 0.016 g L^−1^ day^−1^ under continuous illumination but at higher intensity (140 μmol m^−2^ s^−1^). In those experiments, CO_2_ (2.5 %) was introduced into the algal cultures continuously, which may have contributed to the higher productivity of *S.* *obliquus*. In our study, the biomass productivity of *S. obliquus* under continuous illumination (60 μmol m^−2^ s^−1^) without CO_2_ enrichment was low (0.150 ± 0.006 g L^−1^ day^−1^).

Jacob-Lopes et al. [21] evaluated the growth of the blue-green microalga *Aphanothece* under different illumination cycles (0:24, 2:22, 4:20, 6:18, 8:16, 10:14, 12:12, 14:10, 16:8, 18:6, 20:4, 22:2, and 24:0 (night:day). They found a linear reduction in biomass production with reduction in the duration of the light period, with the exception of the 12:12 (night:day) cycle. Under the conditions of the 12:12 (night:day) photoperiod, the species exhibited higher productivity and maximum cell density than under the other photoperiods applied. Toro [22] reported equal growth rates of the microalgae *Chaetoceros* *gracilis* (a diatom) and *Isochrysis* *galbana* (a haptophyte) under the 0:24 and 12:12 (night:day) regimes; however, the cultures growing at the 12:12 photoperiod were supplied with double light intensity. This implies that the cell growth was also affected by the amount of energy offered per cycle, and not only by the duration of the photoperiod. However, as found for the green microalga *C. vulgaris* [7], the increase in light intensity from 60 to 100 μmol m^−2^ s^−1^ did not exert any positive effect on its biomass productivity.

The varied productivity and growth rate of the algal species investigated in the present work were dependent on the photoperiod and were species specific (over the same taxonomic group Chlorophyceae). The algae studied can be classified into two groups: one growing more efficiently at continuous light (*B.* *braunii* and *S.* *obliquus*) and the second one (3 species of *Neochloris*) growing more efficiently under the 12L:12D regime. These two groups of microalgae differ in the reproduction mode. *B.* *braunii* and *S*. *obliquus* reproduce exclusively by autospores [23], while the species from the genus *Neochloris* reproduce by aplanospores or motile zoospores released from cells in darkness [24, 25]. So far, only *N.* *oleoabundans* has been the object of detailed investigations due to its valuable features [26]. The other unicellular species of the green coccoid algae from the genus *Neochloris* (*N.* *terrestris*, *N.* *texensis*, *N.* *conjuncta*), examined for the first time in this study and exhibiting two modes of reproduction dependent on light conditions, seem to be promising prospective objects for algal biotechnology.
